# BaroFuse, a novel pressure-driven, adjustable-throughput perfusion system for tissue maintenance and assessment

**DOI:** 10.1016/j.heliyon.2016.e00210

**Published:** 2016-12-09

**Authors:** Austin Rountree, Amit Karkamkar, Gamal Khalil, Albert Folch, Daniel L. Cook, Ian R. Sweet

**Affiliations:** a UW Diabetes Institute, Department of Medicine, University of Washington, Seattle, WA, 98195, USA; b EnTox Sciences, LLC, 6901 94th Ave SE, Mercer Island, WA, 98040, USA; c Department of Bioengineering, University of Washington, Seattle, WA 98195, USA; d Department of Physiology and Biophysics, University of Washington, Seattle, WA 98195, USA

**Keywords:** Bioengineering, Pharmaceutical Chemistry

## Abstract

**Objectives:**

Microfluidic perfusion systems are used for assessing cell and tissue function while assuring cellular viability. Low perfusate flow rates, desired both for conserving reagents and for extending the number of channels and duration of experiments, conventionally depend on peristaltic pumps to maintain flow yet such pumps are unwieldy and scale poorly for high-throughput applications requiring 16 or more channels. The goal of the study was to develop a scalable multichannel microfluidics system capable of maintaining and assessing kinetic responses of small amounts of tissue to drugs or changes in test conditions.

**Methods:**

Here we describe the BaroFuse, a novel, multichannel microfluidics device fabricated using 3D-printing technology that uses gas pressure to drive large numbers of parallel perfusion experiments. The system is versatile with respect to endpoints due to the translucence of the walls of the perifusion chambers, enabling optical methods for interrogating the tissue status. The system was validated by the incorporation of an oxygen detection system that enabled continuous measurement of oxygen consumption rate (OCR).

**Results:**

Stable and low flow rates (1–20 μL/min/channel) were finely controlled by a single pressure regulator (0.5–2 psi). Control of flow in 0.2 μL/min increments was achieved. Low flow rates allowed for changes in OCR in response to glucose to be well resolved with very small numbers of islets (1–10 islets/channel). Effects of acetaminophen on OCR by precision-cut liver slices of were dose dependent and similar to previously published values that used more tissue and peristaltic-pump driven flow.

**Conclusions:**

The very low flow rates and simplicity of design and operation of the BaroFuse device allow for the efficient generation of large number of kinetic profiles in OCR and other endpoints lasting from hours to days. The use of flow enhances the ability to make measurements on primary tissue where some elements of native three-dimensional structure are preserved. We offer the BaroFuse as a powerful tool for physiological studies and for pharmaceutical assessment of drug effects as well as personalized medicine.

## 1 Introduction

### 1.1 A need for a high throughput cell/tissue perfusion system

Assessing cell and tissue function is a key task for physiologists and is a critical task for pharmacologists. Many approaches are being taken including cell-based assays of cell viability (such as apoptosis and necrosis), morphology and function [1] (as reflected by the activity of reporter genes [2], proteomics [3] and signaling or regulatory pathways [4]) and a combination of these as in high-content analysis [5]. Many static incubation methods are well suited to high throughput application yet such methods are not capable of high-throughput kinetic measurements—on time scales of minutes, hours and days—on well-maintained tissue with preserved 3D architecture. Dynamic flow-through methods offer a combination of optimal tissue maintenance with assay endpoints yet such methods typically depend on peristaltic or syringe pumps and complex “plumbing” schemes that scale poorly for high-throughput applications. In addition, microfluidics approaches, such as those based on soft lithography, have not provided pharmacologists with adequate and disseminated tools that are practical to use [6]. For such applications as pharmaceutical drug screening or toxicity testing we describe and validate a simplified and scalable solution, the BaroFuse, that we have designed based on our previous flow-through cell and tissue perfusion methods [7], and utilized powerful but easy to implement 3D printing methods [8].

### 1.2 The BaroFuse prototype

We were motivated to develop the BaroFuse in order to scale our current flow-through cell/tissue perfusion methods because using our peristaltic pumps and complex tubing systems scaled so poorly for high-throughput (e.g., to 96 channels) applications. In addition, we were also motivated to lower the flow rates in order to use less media, test compounds, and tissue. We are now replacing the cumbersome peristaltic pumps and perfusion chambers with our BaroFuse prototype that offers stable and very low perfusate flows (1–10 μL/min) driven by gauge pressures between 0 and ≈2 psi of the physiological gas (5% CO_2_, balance air) that overlies the separate perfusate reservoirs. While affording rapid changes of perfusate composition (minutes) experiments can be extended from hours to days while continuously recording a variety of biochemical and biophysical responses.

We have taken an extensible approach with 8-channel BaroFuse modules that can be multiplexed in parallel for high-throughput (e.g., to 96 channels) applications. The BaroFuse is suitable for studying either cultured tissues (e.g., liver slices, pancreatic islets) in microgram quantities, or cultured cells immobilized on, or distributed within a slurry of, culture beads. Here we describe a prototype 8-channel BaroFuse device and its fabrication using stereolithography (a form of 3D-printing), and then validate its function by replicating our prior work for physiological and drug testing. The testing and validation of the BaroFuse were carried out by incorporating oxygen sensing at the outflow, so that the rate at which a tissue sample absorbs oxygen from perfusate flowing through the tissue could be monitored continuously. The measured oxygen consumption rate is an integrated measure of energy generation that reflects changes in cell number, viability and energy utilizing cellular processes, thereby providing an integrated and sensitive reflection of tissue viability and function. We discuss these results in comparison to other tissue incubation/perfusion methods, and by considering BaroFuse technology for high-throughput pharmaceutical drug testing.

### 1.3 Barofuse design requirements

A practical multi-channel cell/tissue perfusion system for physiological and pharmaceutical testing must satisfy, amongst others, the following basic design criteria:

a Design must be modular and extensible—we chose an 8-channel module as our basic building block that is amenable to multiplexing for high-throughput operation.
b Perfusate flow must be pulse-free, invariant from channel-to-channel and stable (less than 2% drift) for hours to days in the range spanning 1–20 μL/min per channel to minimize demand for tissue, perfusate and test compounds, particularly for experiments lasting days.
c Perfusate must be sterile, maintained at 37 °C while physiologically gassed with 5% CO_2_ balance air (for example) to sustain cellular bicarbonate (H_2_CO_3_). Key cell physiological functions are lost when using buffers void of CO_2_ (see Discussion).
d Preparations and operations must be simple so as to allow for repeated use of the system in a time-efficient and reproducible fashion.

## 2 Methodology

### 2.1 The BaroFuse: basic design and fluidic principles

**Fig. 1:**
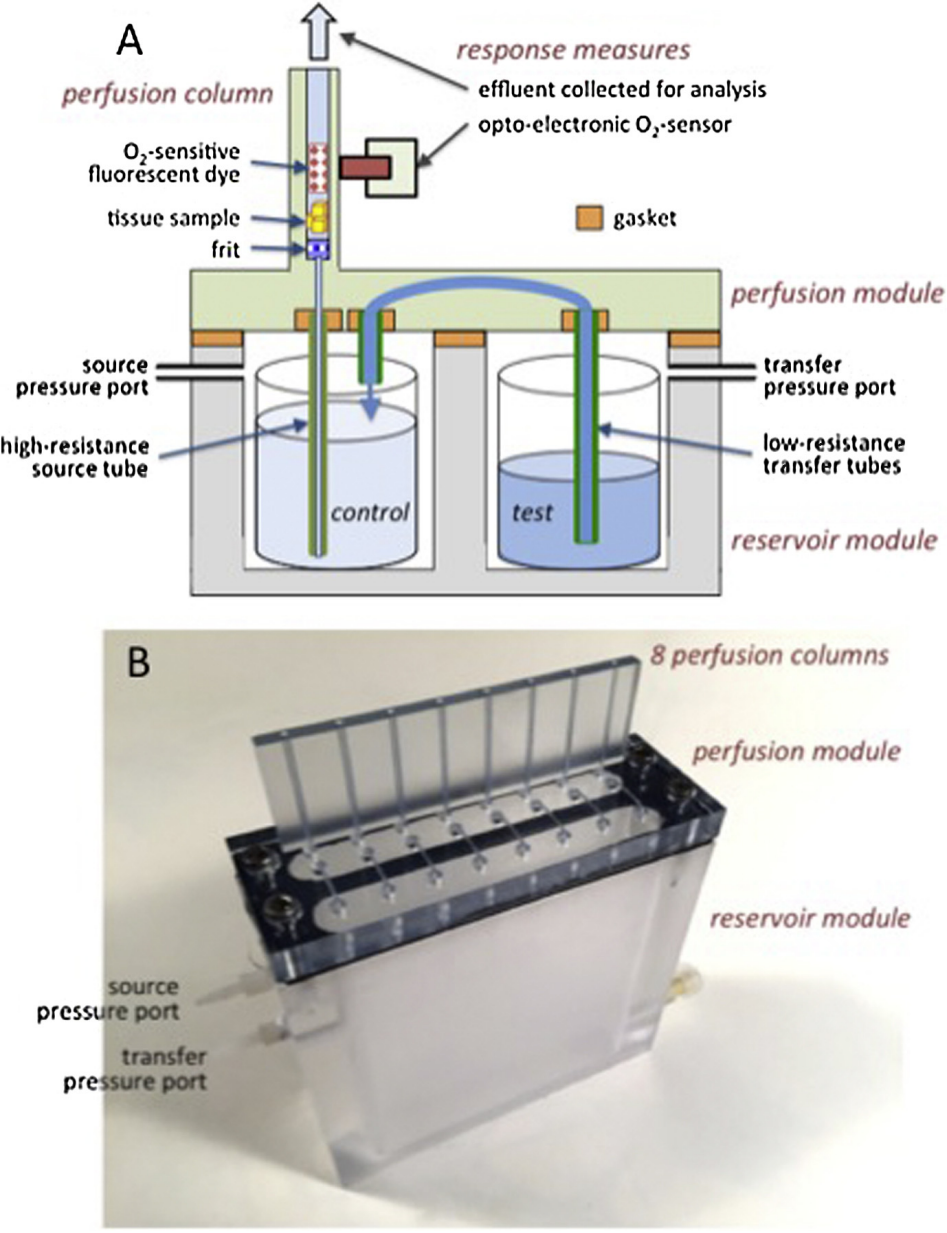
BaroFuse parts and functions. (A) (top) Schematic diagram of the reservoir module topped by the perfusion module that holds the tissue perfusion channels and response measurement. (bottom). (B) An 8-channel BaroFuse prototype, consisting of a *perfusion module* with 8 vertical perfusion columns, is mounted atop a reservoir module adjoined on a (black) silastic gasket. Ports for independently pressurizing source and/or transfer reservoirs are shown at left, while the transfer conduits are visible as the horizontal passages across the septum. High-resistance, low-resistance and transfer perfusate flow tubes are not visible but one of each of these is associated with a perfusion channel and are contained inside the reservoir module.

The BaroFuse is a scalable throughput device for perfusing and assessing tissue samples. The construction and operating principles for a single channel are illustrated schematically in Fig. 1A and our assembled 8-channel prototype used for validation testing is shown in Fig. 1B. Parts of the system are defined in the glossary for reference. Each perfusion channel replicates the geometry and flow characteristics of glass-tube perfusion channels where flow is driven by peristaltic pumps that we have used for numerous published studies [7, 9]. The perfusion chamber (1.5 mm diam.) holds tissue samples (e.g., liver slices or isolated pancreatic islets; see below) or cultured cells on a polyethylene “frit” through which perfusate flows. Samples of the effluent perfusate can be collected for off-line assay—we routinely measure insulin release rate, or lactate production for example. We can also simultaneously measure the tissue’s OCR using opto-electronic sensing of the fluorescence decay rate of an oxygen-sensitive dye painted either on glass beads or on the inner column surface as routinely used in our prior studies [10]. Thus, the BaroFuse can combine conventional chemical and radioimmunological assays with OCR as a powerful index of cellular health and function.

The technical advances offered by the BaroFuse are two-fold. First, the BaroFuse achieves very low perfusate flow rates (e.g., 1–20 μL/min) that are driven in a pulseless manner by the pressure of the physiologic gas (5% CO_2_, balance air) that overlies and equilibrates with perfusate in the reservoirs. Second, perfusate flows can be simultaneously switched from control to test perfusate in all flow channels simply by pressurizing the test compound compartment with a single pressure regulator. In more detail, a “control” perfusate and a “test” perfusate (e.g., drug-containing) are placed in glass test tubes placed in separately pressurized compartments of the reservoir module (Fig. 1A). As described below, experiments are started by pressurizing the source reservoir to fill the flow tubes, and tissue samples are loaded into the perfusion chambers. After a control period, the test perfusate chamber is pressurized sufficiently to drive test perfusate (e.g., drug-containing) across the transfer channel and into the source perfusate reservoir tube thus “doping” the control perfusate with test compounds.

### 2.2 BaroFuse prototype implementation

A Barofuse consists of a lower perfusate reservoir module and an upper tissue perfusion module with a gasket seal at their interface (Fig. 1A). The perfusion module sits atop the reservoir module and contains the tissue perfusion chambers that receive fluid flow from the source reservoir module. Another set of channels mediate the transfer of test compound fluid from the test compound reservoirs to the source reservoirs. The reservoir module is the lower part of the BaroFuse system and is the source of either control- or test-perfusates contained in test tubes in compartments that can be independently controlled to drive flow into perfusion chambers in the perfusion module.

We fabricated the “plumbing” schema in Fig. 1A as a prototype BaroFuse using stereolithography to 3D-print the 8 flow channels into a single perfusion module that includes gasketed insertion points for high- and low-resistance tubes and a transfer perfusate channel (1/16 in. outer diameter, as visible in Fig. 1B). High-resistance source tubes are very small inner diameter PEEK tubing, through which source perfusate flows into the base of a tissue perfusion chamber, driven by pressure in the source reservoir chamber. The inner diameter of the tube, along with the pressure in the chamber, determines the rate of flow in to the tissue perfusion chamber. Low-resistance transfer tubes transfer perfusate containing test compound from the transfer reservoir, through the perfusion module and into the source reservoir. Tissue perifusion chambers are vertical cylindrical channels in the perfusion module that houses tissue while it is continuously bathed in fluid from the reservoir modules from below. The outflow discharges at the top of the chambers, and through which tissue, support beads, and optical sensors are loaded in to the chamber during the setup of the system.

The perfusion module was designed with Autodesk Inventor Pro 2015 software and rendered by stereolithography (Proto Labs, Inc., Maple Plain, MN) in 0.004” layers using a biocompatible (ISO 10993) transparent polymer (WaterShed XC 11122). The reservoir module was machined from clear acrylic plastic, and finished with a 1/16” silastic gasket (GE Premium Silicone II Gasket and Seal) for sealing and isolating the pressurized reservoir chambers, and separate ports for pressurizing (0.5–2 psi) the O_2_/CO_2_/N_2_ gas overlying the perfusate held in 8 test and 8 control test tubes aligned in the reservoir chambers. Tissue effluent can be captured for biochemical assays while perfusion chambers are transparent for optical sensing.

### 2.3 Chemicals and solutions

Krebs Ringer bicarbonate solution (KRB) containing 0.1% BSA, and 25 mM sodium bicarbonate was used for the islet perifusion analyses, prepared as described previously [9]. For the liver perifusions, Williams’ E Media (Sigma-Aldrich, St. Louis, Missouri) supplemented with 10% heat-inactivated fetal bovine serum (Atlanta Biologicals, Lawrenceville, Georgia), 2 mM glutamine, 1% Pen/strep and 20 mM HEPES (Research Organics, Cleveland, Ohio) was used. Glucose, potassium cyanide (KCN), and acetaminophen were purchased from Sigma-Aldrich (St. Louis, MO).

### 2.4 Rat islet isolation and culture

Islets were harvested from Sprague-Dawley male rats (weighing approximately 250 g) (Charles River, Wilmington, MA) anesthetized by intraperitoneal injection of sodium pentobarbital (35 mg/230 g rat). All animal procedures were approved by the University of Washington Institutional Animal Care and Use Committee and all experiments were performed in accordance with relevant guidelines and regulations. Islets were prepared and purified as described [11, 12], and then cultured at 37 °C in RPMI Media 1640 supplemented with 10% fetal bovine serum for 18 hours prior to the experiments.

### 2.5 Mouse liver slice preparation

Liver slices were harvested from male C57BL/6J mice, weighing approximately 20 g (age = 6–8 weeks) (Jackson Laboratory, Bar Harbor, ME) anesthetized by intraperitoneal injection of sodium pentobarbital (3 mg/20 g mouse). All procedures were performed under aseptic conditions in a laminar flow hood. After anesthesia was induced, the midsection was opened up to expose the liver. A piece of liver lobe (size = 4 cm^3^) was removed with surgical scissors. The piece was laid out on a petri dish containing William’s E Media, and after cutting away a layer of capsule, multiple slices were diced (approximately 0.25 × 1 mm (mass = 1–2 mg per piece)) with a scalpel. Two pieces were loaded in to each tissue perifusion chamber for each analysis. After the end of each experiment, the liver samples were weighed. OCR measurements were normalized to this mass.

### 2.6 Measurement of oxygen in outflow

Oxygen tension in the outflow of each tissue perfusion chamber was measured as previously described [10], except that instead of painting the oxygen-sensitive dye on the inside of the tissue perifusion chamber, glass beads (710–1180 microns, Sigma-Aldrich, St. Louis, MO) coated with the dye were layered on top of the tissue in the perifusion chamber. The flow was slow enough so that the beads did not move during the experiment, and the oxygen sensors sampled a representative cross section of perfusate that after passing by the tissue was delivered by convection to the sensors. As previously described, oxygen quenches the amount of phosphorescence emitted by the dye in response to excitatory light, and has a response time of a few seconds [11, 13]. To coat the beads with dye, we submerged them in 100 mL of a dichloromethane solution containing 5 g dimethylsiloxane-bisphenol A-polycarbonate block copolymer (GE, Waterford, NY) and 25 mg of platinum porphyrin (Porphyrin Products, Logan, UT). Coated beads are baked overnight at >100 °C in an oven (Model 1310, VWR, Radnor PA) and the resulting dried crystals are pulverized with a metal spatula. Dye excitation and detection of emitted light from the dye was done via optical fibers, one that carried light from an LED (405 nm) and one that returned emitted light to the spectrometer (MFPF-100 multifrequency phase fluorometer lifetime measurement system Tau Theta, Boulder CO) as previously described [9]. The dye was calibrated by the use of an artificial lung that allowed for rapid changes in oxygen content to be accomplished [9], and the slope of the signal was 2.2 μs lifetime/(35 nmol O_2_/ml). Rather than continuously measuring the inflow concentration of oxygen, we determined it by temporarily altering the flow rate, typically by doubling it, and calculating the inflow oxygen concentration using for each 2 flow rates (FR) in the equation OCR = FR(O2_in_–O2_out_), and then solving the resulting 2 equations for O2_in_.

### 2.7 Analysis of outflow fractions

Fractions were collected by use of a FOXY R2 (Teledyne Isco, Inc., Lincoln, NE) to collect outflow in to a 96 well plate. Flow rate was calculated by weighing the contents of each well, and then dividing the measured mass by the time interval of collection. Insulin in the outflow fractions was measured using an RIA kit (Linco Research Inc., Billerica, MA). Trypan blue concentration in the outflow was measured by absorption at 604 nm in a Synergy 4 microplate reader (BioTek, Winooski, VT).

### 2.8 Procedures for perfusion experiments

For experiments, eight 13 × 100 mm test tubes (9 mL) are inserted into each side of the reservoir chamber, and each filled with a test or control perfusate. Eight high-resistance (i.e., small-bore) and eight low-resistance (i.e., large-bore) flow tubes, ≈100 mm long, are inserted into gasketed ports at the base of the perfusion module, and a short, large-bore tube is inserted to complete the perfusate transfer path by which test substances are transferred from the test compound test tube and diluted to the desired experimental concentration in the “control” source test tube. The inserted gaskets are cut from PharMed BPT tubing (Cole-Parmer Instrument Co., Vernon Hill IL). To control the temperature of the system, a thermostated immersion circulator/heater (model 1122S, VWR, Radnor, PA) regulated a plexiglass box (17 × 17 × 13 (w × d × h) inches) filled with water, into which the BaroFuse system is submerged.

We placed 8 test tubes into each side of the fluid reservoir block and then filled the source tubes with control fluid, and the test compound perfusate tubes with fluid containing the selected test compounds at the desired composition. The tissue perfusion block was then placed on top of the fluid reservoir block and screws tightened. Tubing (Masterflex L/S 16 tubing, Cole-Parmer Instrument Co.) was attached to the two source pressure ports on the fluid reservoir block and secured without yet hooking these tubes up to the pressure regulators, and the device was partially submerged into the water to a depth that allowed the top 1 inch of the fluidics systems to protrude. The immersion heater was turned on for 30 minutes until the water temperature reached 37 °C. Then reservoir gas tubes were hooked up to pressure regulators set to the desired pressure (0.5–2 psi) and both fluid reservoir block chambers were purged with the 5% CO_2_/balance air for 5 minutes. Once the vent ports are capped, the tissue perfusion chambers filled up with perfusate, the perfusate was collected, and the flow rate was confirmed or adjusted as needed. Next, tissue was loaded in to the tissue perfusion chambers and allowed to settle to the bottom of the chamber. If islets or cells were used, a porous frit was first inserted in to the perifusion chamber and pushed to 0.5 cm from the bottom of the chamber prior to adding the islets. Finally, outflow tubes (1/16th inch OD tubing (HPFA, IDEX)) were inserted into the tissue perfusion chambers to allow submersion of the device beneath the surface of the water in the water bath and collection of outflow if desired. For a typical experiment, sampling of the effluent and recording control pO_2_ levels proceeded for 2–3 hours to establish a stable baseline before transferring “test” solutions into test tubes in the source reservoir. At this time, we transiently (10–15 s) pressurize the test reservoir to >2 psi to inject the contents of test compound reservoirs through the transfer channel and into the still-flowing control perfusate.

## 3 Results

### 3.1 Overview of validation

Calculating the increment or decrement of substances (e.g., oxygen, hormones, metabolites) either extracted or released by the tissue demands that the perfusate flow rate be known and stable. We first characterized the control and stability of flow rates and their dependence on reservoir pressure and tubing resistance for extended periods of time. We then validated the BaroFuse for biological testing tasks by replicating our prior results showing changes in OCR in response to increased glucose level by a small number of isolated islets, and to the drug acetaminophen by precision-cut slices of rat liver tissue.

### 3.2 BaroFuse functional validation

**Fig. 2:**
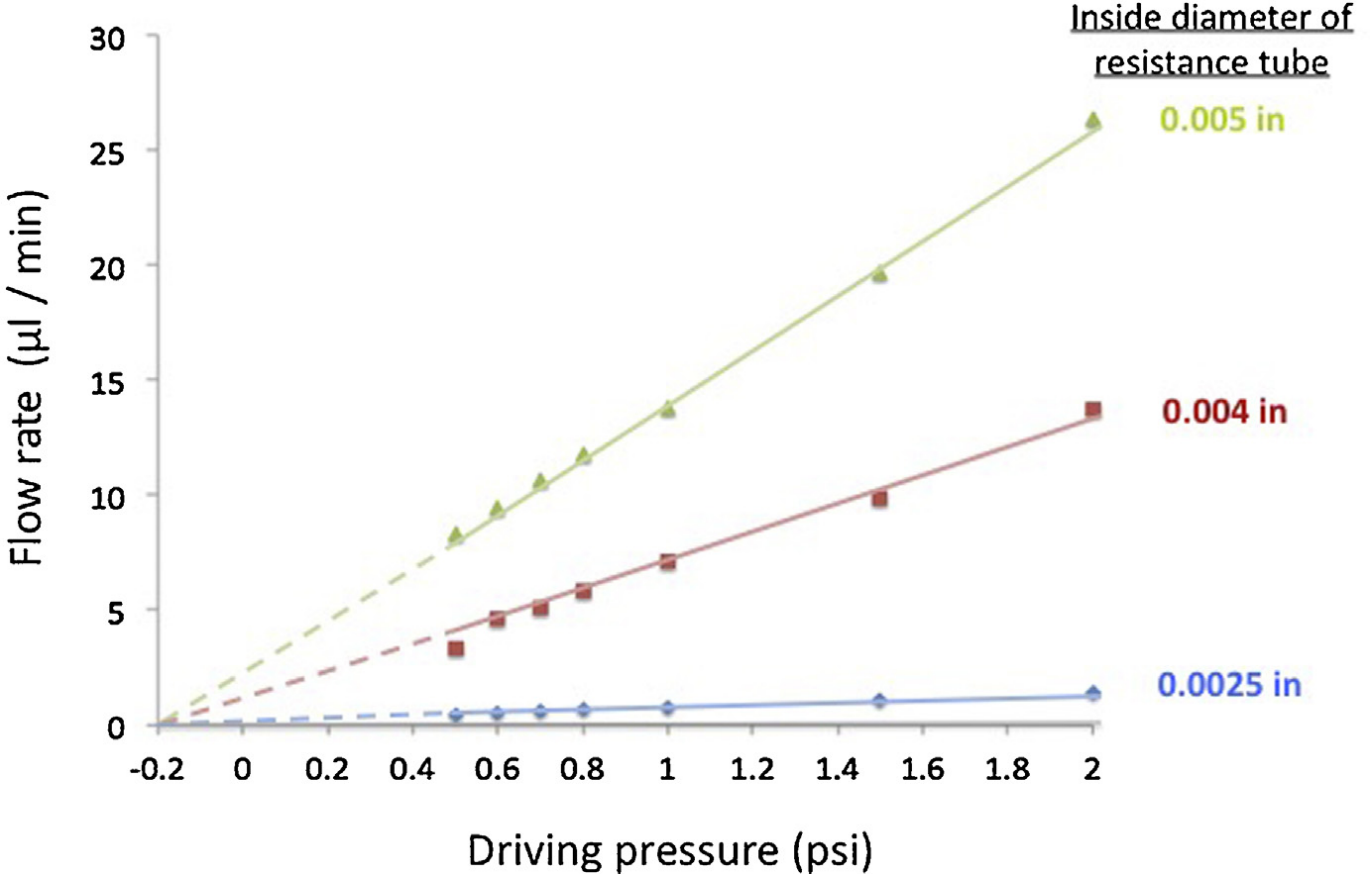
Pressure-flow rate test confirms ability of the BaroFuse to control flow rates. Flow rates were measured for different pressures generated by pressurized gas using 3 different resistance tubes with inner diameters as indicated. Pressure was changed by adjustment of the pressure regulator. Lengths of resistance tubes were all ≈ 100 mm. Due to gravitational forces, the flow continues even when there is no added pressure from the gas tank, and would only become 0 if negative pressure of −0.2 psi would be induced.

The key to the BaroFuse design and function is that perfusate flow in all channels is controlled directly by changing the gas pressure (0.5–2 psi) in the reservoir chamber and very low and stable rates of flow are achievable. Fig. 2 demonstrates that high-resistance flow tubes (1/16” OD PEEK tubing (IDEX, Lake Forest, IL)) with inside diameters of 0.0025”, 0.004” or 0.005” provide the required resistance to produce the desired range of flows. Such small-bore tubes contribute such high resistance that flow resistance from the reservoir to the perfusion chamber is determined by the resistance tube, with little contribution from the larger flow passages (ID > .025 in). Perfusate flow was collected and measured at each of several perfusate reservoir pressures from 0.5 to 2.0 psi and was finely controllable over a range of <1 to 20 μL/min as desired for practical tissue perfusion tasks. Thus we achieved our design goal that flow rate is linearly related to driving pressure as expected of laminar (non-turbulent) fluid flow which depend on the fourth-power of tube diameter as interpolated (the lines) according to the Hagen–Poiseuille equation (F is proportional to ΔP x R^4^/L, where F is the flow rate, P is pressure, R is the tube radius and L is the tube length). Furthermore, extrapolation of the these lines shows that they intersect a common, no-flow pressure of −0.2 psi that corresponds exactly to the back-pressure exerted by the 5.5-in column of perfusate fluid reservoir tubes and the top of the flow columns.

### 3.3 Tracking a change of perfusate composition

Flow rates were measured before, during and after the transfer of media from the test source reservoirs. At the desired time, the pressure in the transfer pressure reservoir was increased to 2.5 psi and the media had completely moved in to the test tubes in the source reservoirs within 15 seconds, at which time the test compound pressure regulator was shut back off. No fluctuation in flow rate was observed in the outflow (data not shown). In order to assess the time it took for the tissue chamber to be exposed to the second media composition, trypan blue (0.008%) was added to the test tube in the second compartment and its concentration in the outflow was measured by light absorbance. The actual time it takes for equilibrium to be reached, and the final composition of the outflow after mixing, depend on the initial contents of the test tubes in the two chambers. When the volumes in the source reservoir and the transfer reservoir were equal, it took 15 minutes for the outflow concentrations to reach steady state, (data not shown). The concentration in the outflow at steady state was simply the weighted average of the composition with respect to volume. More rapid changes in media composition could be obtained by transferring a larger amount of media in the transfer reservoir to a small amount of media in the perfusion reservoir.

### 3.4 Measuring islet OCR in response to glucose stimulation

**Fig. 3:**
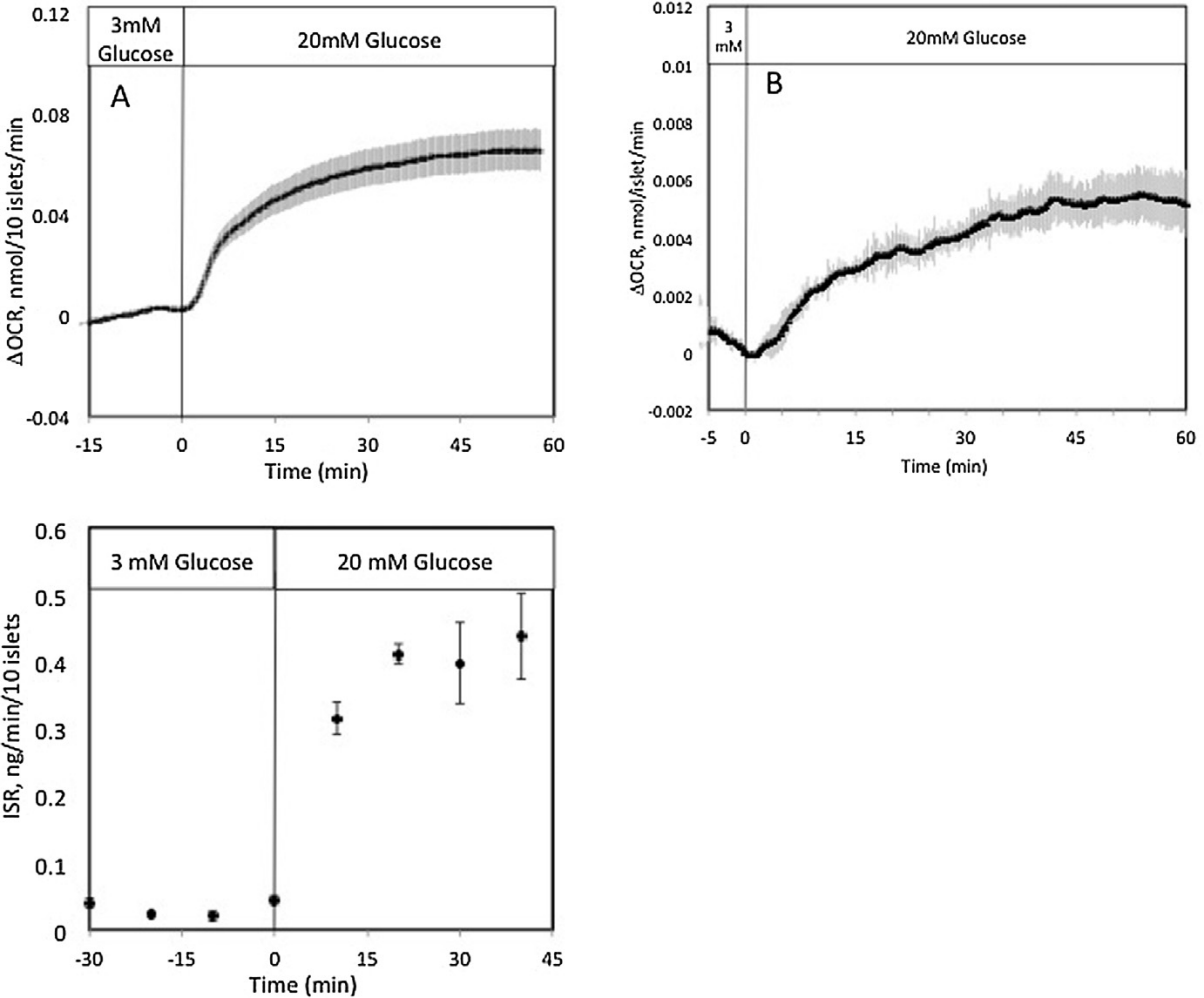
Functional response of pancreatic islets in the BaroFuse. (A) (top) OCR by 10 isolated rat islets was measured in response to glucose simultaneously in 3 of the 8 channels (flow rate was 7 μL/min). (bottom) Outflow fractions were collected in three of the channels and assayed for insulin. (B) OCR by 1 isolated rat islet was measured in response to glucose simultaneously in 3 of the 8 channels (flow rate was 1.5 μL/min). Data for both curves are averages ± standard error of the mean (n = 3).

To develop a system to assess islet function that is expandable to a large number of channels, it is especially important to miniaturize the system to minimize the number of islets required to produce a detectable endpoint. In the case of OCR measured as the difference between inflow and outflow oxygen concentration, the detection limit is linked to the number of islets and the flow rate. The lower the flow rate, the lower the number of islets that are required. Islets (10/chamber) were perifused at 7 μl/min, and OCR was stable, and the variability was only a few percent of the glucose responses (Fig. 3A). The effects of glucose were very similar for all channels. At the end of the experiment, the flow rate was doubled (by doubling the pressure) two times and the actual OCR was calculated as described in the methods (change in OCR = 0.63 ± 0.082 nmol/100 islets/min) in response to 20 mM glucose). Outflow fractions were collected and the content of insulin was measured. Both OCR and insulin secretion responses to glucose were consistent with previous, larger scale perifusion systems [7]. To determine if the system could resolve glucose responses with very small amounts of tissue, 3 channels were loaded with only 1 islet, and a flow rate of 1.5 μL/min was selected (using a high resistance tube of 0.0025” ID, and pressure of 2 psi). Again, glucose responses were well-resolved and similar to that obtained with higher numbers of islets, although the data was noisier than that obtained with 10 islets (Fig. 3B). These examples demonstrate the ability of the system to generate 6 real time OCR responses with only 60 islets, and even less if only steady state changes are needed.

### 3.5 Response of hepatic OCR to acetaminophen

**Fig. 4:**
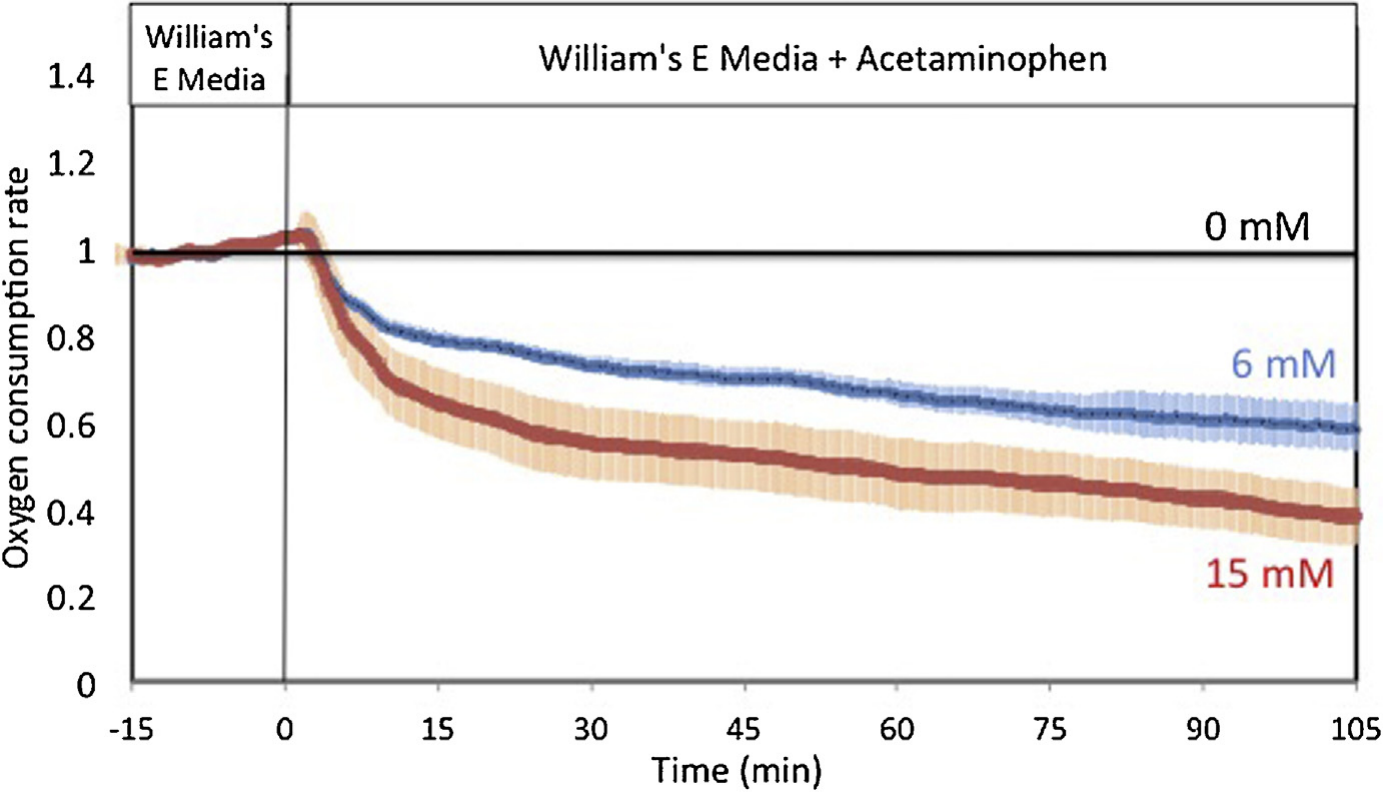
Effect of acetaminophen on liver oxygen consumption in the pressure-driven micro-perifusion system. OCR by 1 mg slices of mouse liver was measured in response to acetaminophen. Data are plotted as averages ± standard error of the mean (n = 3).

To support the utility of the BaroFuse to the pharmaceutical industry, we replicated our prior characterization of the effects of the drug acetaminophen on liver OCR. We cultured liver slices overnight, loaded two 1 mg liver slices into each tissue perfusion chamber and perfused them for 3 hours to measure baseline OCR. We then switched six channels to acetaminophen-containing perfusates (2 concentrations in triplicate) and were able to demonstrate the reproducibility of OCR inhibition (Fig. 4). OCR in the two channels that were not exposed to acetaminophen remained flat, whereas the OCR was lowered 40 and 60% by 6 and 15 mM acetaminophen. These results were similar to those obtained in a previous study with our larger peristaltic pump-driven perifusion system [10].

## 4 Discussion

### 4.1 Features of the BaroFuse high throughput perifusion system

It is well recognized that flow systems offer distinct advantages over non-flowing systems for maintaining and assessing cell function [13, 14]. Oxygen delivery is better, especially for tissue and multi-cellular structures, and flow allows for exact determination of consumption or production of chemical entities (based on the conservation of mass as the difference between the contents of the outflow minus the inflow). Thus, we measured OCR with very high sensitivity and high run-to-run reproducibility so we could record the dynamic responses to test compounds in ways not available with static incubation systems. A key BaroFuse feature is that the pressurized gas that drives perfusate flow contains 5% CO_2_ so that BaroFuse perfusates are pH-buffered physiologically. This is important because bicarbonate/CO_2_ buffering is essential for a variety of biochemical functions including gluconeogenesis [15], fatty acid synthesis [16], insulin secretion [17, 18], and the citric acid cycle [19] as well as for cell growth and replication [20, 21]. In addition, the near physiological, flow-through conditions enable experiments of long duration (hours to days) and can be used for studying both acute and slow drug effects.

### 4.2 Ease of use, the technical challenge

We seek to develop a continuous perfusion method that is easy-to-use, reliable and scalable for high-throughput applications. The BaroFuse design is modular and consists of only two mating parts, and flow in all channels is accomplished by a single gas regulator that controls the pressure in the reservoir modules as described. Since the assembly time of the system is brief, tissue and perfusate preparation and allocation accounts for most of the set up time, and it took about 20 minutes to load the tissue in to the 8 channels and fill the chambers with the selected media. Although we did not directly scale our system up from 8 channels, our system is modular and through put can readily be increased by placing multiple devices next to each other. Future versions will incorporate higher numbers of channels in to a single 3D printed module, and we estimate that throughput can be increased significantly with a 2–4 hour set up time.

### 4.3 BaroFuse miniaturization

By miniaturizing the BaroFuse for high-throughput tissue testing, we have significantly reduced the requirement and expense of a) isolating large amounts of tissue and b) preparing large amounts of drug-containing perfusates. For example, a typical study of OCR by perfused islets required 500–750 islets per channel with perfusate flows of 50–100 μL/min [7, 22] while static incubation systems such as SeaHorse require 50–80 islets per well [23]. However, we have used the BaroFuse for robust and reproducible OCR measurements using only 1–10 islets per channel which is very practical for high-throughput studies given the islet counts in a mouse (n ≈ 250) or rat (n ≈ 750). Thus, a 96-channel experiment could be carried out with a few hundred islets, obtainable with a single mouse or rat. In its current configuration, we tested stable flow rates of 3 μL/min that would run for 48 h from a 9-mL perfusate reservoir, or much longer simply by increasing the capacity of the perfusate reservoir module.

### 4.4 Options for assessing functional endpoints

We illustrated the use of the BaroFuse by measuring OCR using highly sensitive sensors yet other sensors and assay can be applied to the BaroFuse technology. Whereas we demonstrated here optical detection of OCR, we have previously used optical fluorescence measure of the cell or tissue to directly assay cellular NAD(P)H and calcium as well as genetically expressed sensors for H_2_O_2_ and ATP. Furthermore, we envision that the BaroFuse design can be adapted to the practical implementation of a perfusate fraction collector method by which hormones, metabolites and signaling molecules can be measured simultaneously with the above optical methods.

### 4.5 Comparison of BaroFuse with other fluidic approaches to assessment of intact tissue

We have designed the BaroFuse system with specific operational requirements in mind that are not easily implementable with previous approaches to microfluidics. For instance, microfluidic systems are historically made by lithography out of materials such as polydimethylsiloxane (PDMS) that are gas permeable [24], and in some cases this property is utilized for controlling oxygen levels in the microfluidics device [25, 26]. However, gas permeability also makes it difficult to measure OCR although oxygen tension can be measured [27]. Resins used with stereolithographic 3D printers such as the WaterShed XC 11122 formulation used in our fabrication are gas impermeable.

Very sophisticated microfluidics systems can be configured with a high number of channels, and with flow control by multiple microvalves [28, 29]. For instance, a single tissue sample has been treated sequentially with many different drugs [30]. However, the goal of having a large number of tissue perifusion chambers each with its own perfusate supply to facilitate the testing of for instance 96 drugs on 96 separate tissue samples using lithography has not been previously developed. The BaroFuse has a macrosystem for media reservoirs that are easy to load and can contain required amounts of gas-equilibrated media for extended protocols.

Assessing effects of drugs on tissue with respect to OCR and cellular acidification (a combined reflection of CO_2_ and lactate production) has been carried out with two commercially available systems, one with a 6-channel stop flow analysis (Bionas 2500 analyzing system [31]) and a multi-well static system designed for monolayers of cells (SeaHorse XF Extracellular Flux Analyzer, [32]. Fluidics systems are positioned to study interorgan relationships, for instance as was carried out with a two-microchamber biochip to investigate the interaction between intestine and liver [33]. Future changes in the 3D printed plumbing for the BaroFuse could readily facilitate perfusion of tissue chambers in series.

### 4.6 Summary and conclusions

We have introduced, described and evaluated BaroFuse, a flow-through tissue perfusion method that is based on our prior work and is scalable for high-throughput, multichannel operation for physiological and pharmaceutical applications. The BaroFuse is based on a simple principle applied in a novel fashion so that very small tissue samples can be perfused at very low flow rates by simply replacing the mechanics of peristaltic pumps by increments of the gas pressure in perfusate reservoirs. We have implemented this simple, but novel, approach using state-of-the-art 3D-printing of our extensible fluidic device. We anticipate that the BaroFuse can be deployed and used in a broad range of laboratory settings that require low- or high-throughput testing of tissues exposed to physiological, pharmacological and toxicological substances and application in personalized medicine.

## Declarations

### Author contribution statement

Austin Rountree: Performed the experiments; Analyzed and interpreted the data.

Amit Karkamkar, Gamal Khalil, Albert Folch: Contributed reagents, materials, analysis tools or data.

Daniel L. Cook, Ian R. Sweet: Conceived and designed the experiments; Analyzed and interpreted the data; Wrote the paper.

### Competing interest statement

The authors declare the following conflicts of interest: I.R.S, D.C. and G.K. have stakes in the company EnTox Sciences that participated in the project. A.F. and A.K were remunerated by EnTox, but have no stakes in the company.

### Funding statement

This work was funded by grants from the University of Washington CoMotion Innovative Fund (F2015_7071_Sweet), and the NIH (P30 DK17047 (Cell Function Analysis Core), NCATS R41 TR001196).

### Additional information

No additional information is available for this paper.
